# Visible region absorption in TMDs/phosphorene heterostructures for use in solar energy conversion applications

**DOI:** 10.1039/d0ra05810f

**Published:** 2020-08-26

**Authors:** Ashraf Maniyar, Sudhanshu Choudhary

**Affiliations:** National Institute of Technology Kurukshetra India ashrafchand14@gmail.com hellosudhanshubit@gmail.com sudhanshu@nitkkr.ac.in +917206550867

## Abstract

Heterostructures of pristine black phosphorene (P) with transition metal dichalcogenides (TMDs) monolayers of MoS_2_, MoSe_2_, MoTe_2_, WS_2_, and WSe_2_ are investigated using density functional theory based simulations. The results suggest that individual MoS_2_, MoSe_2_, MoTe_2_, WS_2_, WSe_2_, and black phosphorene have high absorption in some portions of the visible region (∼390–430 nm) and in the entire ultraviolet (UV) region. All the heterostructures results into redshift phenomena where absorption peaks are seen to shift to lower energies of the spectrum. The absorption coefficient is seen to increase with the wavelength and appears to be shifted towards the red end of the spectrum. High absorption is also observed in the entire visible region (*λ* ∼ 410 to 780 nm) of the spectrum for all heterostructures. This high absorption in the desired visible range may find many potential applications for the heterostructure, such as in the fabrication of optoelectronic devices and solar cells. The refractive index and dielectric constant of the heterostructure are also calculated and are found to be in line with trends in dielectric constant. Furthermore, it is observed that most of the resultant heterostructures have type-II band alignment which is ideal for solar energy conversion and optoelectronic applications.

## Abstract 2

Heterostructures of pristine black phosphorene (P) with transition metal dichalcogenide (TMD) monolayers of MoS_2_, MoSe_2_, MoTe_2_, WS_2_, and WSe_2_ are investigated using density functional theory based simulations.

## Graphical abstract

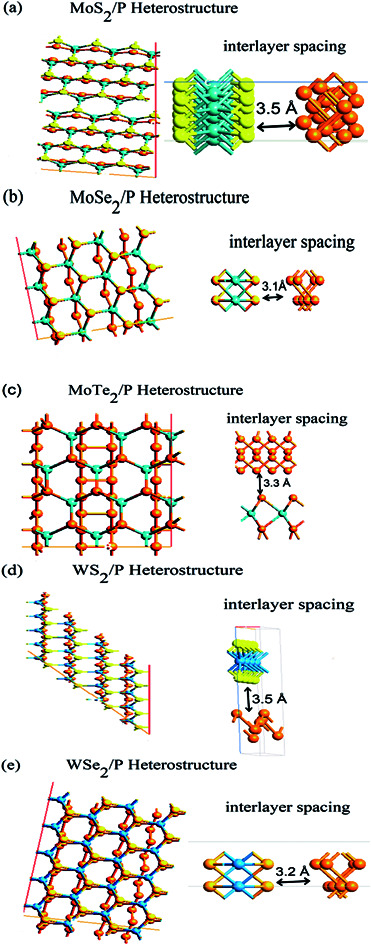

## I. Introduction

Two-dimensional (2D) monolayer graphene which was experimentally discovered in 2004, has created huge interest among researchers for conducting more studies on 2D nanomaterials as they possess distinctive physical and chemical properties which can be used in various applications in the field of optoelectronics and nanoelectronics.^1–6^ Since then, other two dimensional materials that have unique properties have been explored such as germanene,^7^ silicine,^8^ phosphorene^9^ and transition metal dichalcogenides (TMDs) like MoS_2_, MoTe_2_, MoSe_2_, WS_2_, and WSe_2_.^10^ These layered 2D substance have many of the properties of graphene and find use in electronic devices. For example, to design an excessive performance field-effect-transistor (FET), germanene is usually used inside the device channel. Due to its novel electronic characteristics, hexagonal boron nitride (h-BN) presents a perfect 2-D structural copy of graphene and because of its large insulator type bandgap of ∼6.3 eV it acts as a terrific dielectric substrate.^11^ However, the insignificant bandgap of graphene limits its use in optical applications and hence we focus our interest on TMDs. Phosphorene is flexible like graphene and it can be mechanically desquamated. Graphene is rarely used in optoelectronic applications due to the absence of a bandgap whereas, monolayers of all TMDs have indirect or direct bandgaps that can be tuned by varying the number of layers which makes TMDs an ideal material for optoelectronic applications.^12–15^

While there exist many single and bilayer materials with weak van-der-Waals interaction between the layers and strong in-plane covalent bonds.^16^ Materials like TMDs (MoS_2_, MoSe_2_, MoTe_2_, WS_2_, and WSe_2_) have received a remarkable interest because of their unique optical and electronic properties. It is a known fact that the bandgap changes (from indirect to direct) when a single layer is exfoliated from a bilayer material.^17^ TMDs are also composed of van-der-Waals bonded X–Mo–X and X–W–S (X = S, Se, Te) units like graphene.^18–20^ To obtain ultrathin crystals of TMDs, a micromechanical cleavage technique can be used, as the bonds between two different layers in MoS_2_, MoSe_2_, MoTe_2_, WS_2_, and WSe_2_ crystals are very weak whereas it has strong interlayer interaction.^21^ Previous research works have suggested that the properties of MoS_2_, MoSe_2_, MoTe_2_, WS_2_, and WSe_2_ can be tuned by varying the number of layers^12,13^ and can thus find application the field-effect transistors and optoelectronic devices.^17,21–25^

Optical absorption in heterostructures consisting of atomically thin TMDs strongly depend on the phase, structure and thickness of the TMD layers.^26^ The dispersion energy, optical spectra moments and the dielectric constant were all found to decrease with increase in SnS thin film's thicknesses.^27^ The number of layers (thickness) can manipulate the electronic properties of Mo and W based TMDs, where an increase in bandgap was observed with decrease in thickness from bulk to monolayers.^28^ Energy gap and refractive index were reported to vary with thickness of CdI_2_ films in ref. 29. An increase in refractive index with thickness of CdSe thin films was reported in ref. 30. In ref. 31 a blue shift in the optical bandgap was observed with respect to the bulk value on increasing the thickness of ZnO thin film deposited on Si. Dependence of optical properties on the thickness of amorphous Ge_30_Se_70_ thin films is investigated in ref. 32, where it was reported that increasing the film thickness increases the direct optical energy gap and decreases the refractive index. Heterostructures consisting of atomically thin TMDs exhibit excellent electronic and optoelectronic properties and great potential in optoelectronics and photovoltaics as optical absorption strongly depend on the phase structure and thickness of the TMDs layer. The single layers of TMDs can be used in the fabrication of high mobility FET.^33^ The TMD heterostructure with black phosphorene can show dramatic improvements in its optical properties. P–n diode photodetector made up of heterostructure of MoS_2_ and black phosphorene has shown high responsivity of 418 mA W^−1^, which is approximately 100 times more than the phototransistor of black phosphorus.^34^ MoS_2_ heterostructure with AlN and GaN have been suggested for ultra high effectiveness in the field of photocatalytic activity that can be useful for water splitting application.^35,36^

Phosphorene is a p-type semiconducting material which is abundantly available in nature and is a popular material.^37^ Monolayer phosphorene has unique material properties like graphene and MoS_2_, and it can be mechanically exfoliated.^38^ It has well-built physical anisotropy and high conductance that finds applications in the field of nano-electronics and optoelectronics.^39^ Not only multi-layered but single-layered black phosphorene have also been successfully exfoliated.^9,40–42^ Previous research works have shown that black phosphorene has good carrier mobility and has direct bandgap.^43–45^ Phosphorene has a puckered structure in which each phosphorus atom is bonded covalently with the other three atoms. Phosphorene material has turned into one of the most researched hotspots due to its excellent properties which can be used in optoelectronics and sensor applications.^46–48^

Nowadays, almost all the layered 2D materials have attracted the researchers because of their usefulness in optoelectronics and photonics applications, their heterostructures have shown outstanding optical properties. The fabrication of heterostructures are possible by stacking 2D materials technique.^49^ Two n-type monolayers of MoS_2_ stacked on P-type WSe_2_ forms a p–n junction in which type-II band alignment exists.^50–52^ Heterostructures of phosphorene with graphene and boron nitrides have been investigated using images obtained from scanning tunneling microscopy (STM).^53^ Wearable batteries, distinct types of energy harvesters and supercapacitors and other wearable energy sources based on 2D materials have been suggested in ref. 54. Moreover, heterostructures of MoS_2_-on-MXene can be accomplished in batteries that are made using Li-ion due to the enhanced Li_2_S and Li adsorption and associated metallic property.^55^

2D materials have different energy band structures and bandgaps. However, monolayers of MoS_2_, MoSe_2_, MoTe_2_, WS_2_, WSe_2_, and phosphorene have almost similar bandgap with similar hexagonal structure^56^ and are potential materials for light accumulation. Phosphorene has high carrier mobility, while MoS_2_, MoSe_2_, MoTe_2_, WS_2_, and WSe_2_ have high photo responsivity. It would be interesting to investigate their heterostructures that could display desired band alignment for ruling out the recombination of electron–hole pairs.

It is understood that there exist many studies on phosphorene/TMD heterostructures with type-II band alignment,^20,34,42,56^ however, most of them lack detail investigations on electronic and optical properties. Some of them have reported only the electronic structure with a small set of TMDs, and the others have reported only optical properties of blue phosphorene/TMD heterostructures. In this paper, we perform a detail investigation on the heterostructures of black phosphorene with all possible set of suitable TMDs and have included the results on binding energy calculations for experimental stability, band structure calculations for electronic property, and band alignments, refractive index, dielectric constant and absorption coefficient calculations for predicting optical properties. The results suggest that the absorption in black phosphorene can be tuned to higher levels due to red-shift or shifting of absorption peaks to lower energies in the black phosphorene/TMD heterostructure. It is found that MoS_2_/P MoSe_2_/P, MoTe_2_/P, WS_2_/P, and WSe_2_/P heterostructures result in large absorption in the visible and IR regions due to combined properties of MoS_2_, MoSe_2_, MoTe_2_, WS_2_, WSe_2_, and phosphorene. Potential applications of the heterostructures could be their use as photovoltaic materials. The resulting electronic properties also suggest that the heterostructures demonstrate type-II band alignments, hence constraining the photo-generated recombination of electron–hole pairs. The 2D and layered materials have considerable properties from their bulk crystals and find application in energy storage devices and electronics.^57,58^

## II. Computational details

**Fig. 1:**
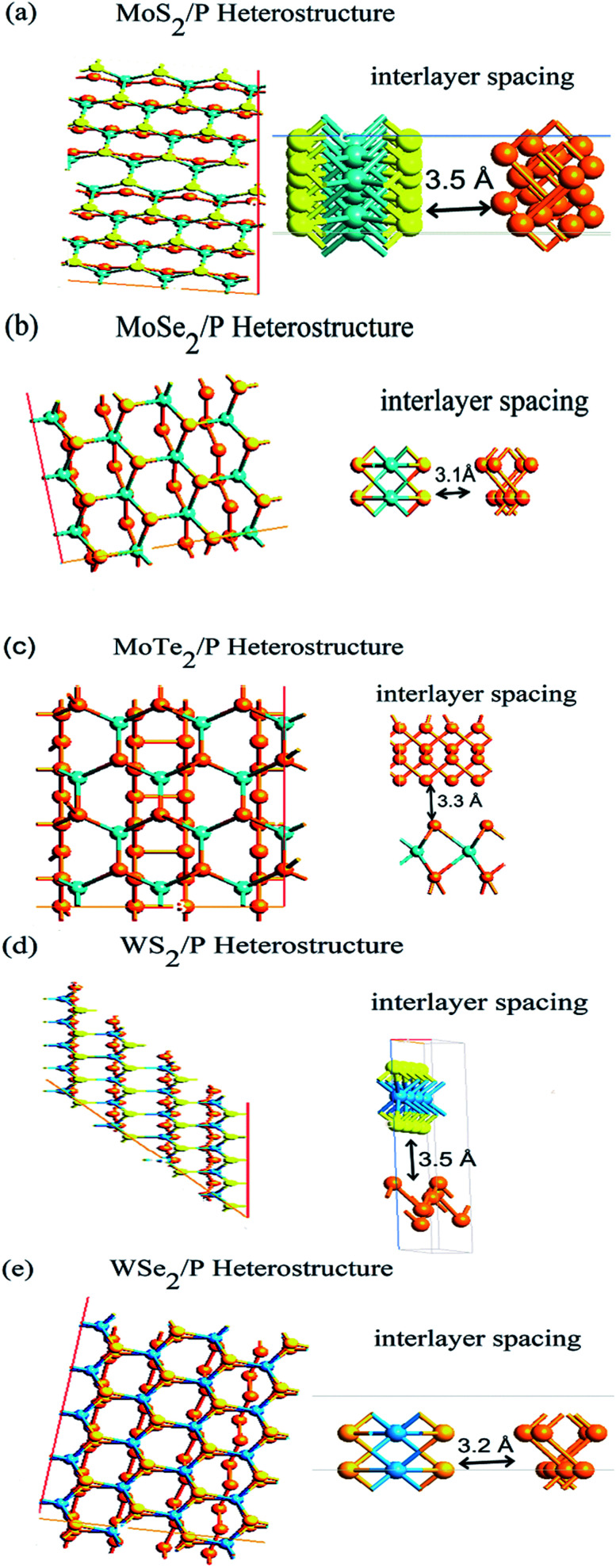
Optimized structures of (a) heterostructure of MoS_2_/P, (b) MoSe_2_/P, (c) MoTe_2_/P, (d) WS_2_/P, (e) WSe_2_/P, with their interlayer distances with phosphorene.

The optical as well as electronic properties are studied for MoS_2_/P, MoSe_2_/P, MoTe_2_/P, WS_2_/P, and WSe_2_/P heterostructures. Thickness of MoS_2_, MoSe_2_, MoTe_2_, WS_2_, WSe_2_ and phosphorene are 0.65 nm, 0.7 nm, 32 nm, 1 nm, 0.7 nm and 2.1 nm, respectively. Number of atoms in MoS_2_/P, MoSe_2_/P, MoTe_2_/P, WS_2_/P, WSe_2_/P heterostructures are 40, 20, 34, 20 and 20, respectively. Simulations were performed using first-principles method based on density functional theory (DFT) on Atomistix Tool Kit (ATK). The exchange-correlation energy of the system is approximated using generalized gradient approximation (GGA) of Perdew–Burke–Ernzerhof (PBE).^59,60^ For geometry relaxation of all atoms, LBFGS algorithm with force tolerance 0.05 eV Å^−1^ is used. Optimized structures are shown in Fig. 1. Brillouin-zone sampling with 9 × 9 × 1 *k*-points in *x*–*y*–*z* direction has been used, mesh cut-off energy is set as 150 Ry.^50^ Semi-empirical DFT-D2 Grimme method was used since standard PBE function cannot completely describe van der Waals (vdW) interactions.^61^

For obtaining optical properties, DFT with *meta*-GGA is used with 15 × 15 × 1 *k*-point brillouin zone sampling as in ref. 62. Considering thermal effects on the optical properties of the structure, broadening is set as 0.05 eV.

The calculation of susceptibility tensor is performed with the help of Kubo–Greenwood equation as
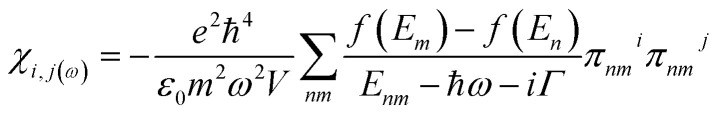
where *π*_*nm*_^*i*^ is the *i*^th^ component of the dipole matrix element between state *m* and *n*, *Γ* is the broadening, *f* is the Fermi function and *V* is volume.

For calculation of susceptibility, relative dielectric constant ‘*ε*_r_’, response coefficients, optical conductivity ‘*σ*’, and polarizability ‘*α*’ are used as^63^*ε*_r_ = (1 +*χ*(*ω*)),*α*(*ω*) = *Vε*_0_*χ*(*ω*), *σ*(*ω*) = −img(*ωε*_0_*χ*(*ω*))

The refractive index *η* is defined in terms of *ε*_r_ and extinction coefficient *κ* as:
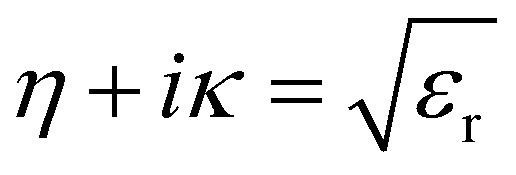

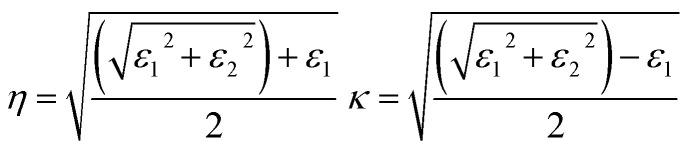


Coefficient of optical absorption is related to the extinction coefficient as^64^
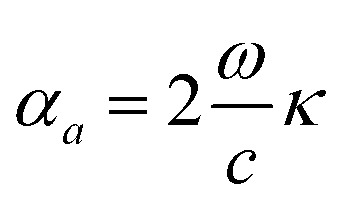


## III. Results and discussions

### A. Geometric structure and stability

The structural parameters of monolayer MoS_2_, MoSe_2_, MoTe_2_, WS_2,_ WSe_2_, and pristine black phosphorene are inspected to establish an appropriate heterostructure. Graphene has a planar structure,^6^ whereas, phosphorene has honeycomb puckered structure in the armchair (*y*-axis) and bilayer in the zig–zag (*x*-axis). The in-plane P–P bonds length is 2.22 Å and the interlayer bridge length is 2.24 Å. The in-plane bond angles are 96.34° and out of the plane bond angle is 102.09°. The MoS_2_, MoSe_2_, MoTe_2_, WS_2_, and WSe_2_ monolayers consist of weakly bonded S–Mo–S, Se–Mo–Se, Te–Mo–Te, S–W–S, and Se–W–Se structures, respectively. The in-plane lattice parameters are calculated as *a* = 3.16 Å for MoS_2_, *a* = 3.28 Å for MoSe_2_, *a* = 3.51 Å for MoTe_2_, *a* = 3.15 Å for Ws_2_, and *a* = 3.28 Å for WSe_2_, with *γ* = 120° which are in confirmation with other studies.^17,40^

The optimized heterostructures are shown in Fig. 1, which are developed in a way that produces minimum lattice mismatch. The lattice mismatch values obtained for the heterostructures are 3.69%, 3.32%, 3.48%, 3.33%, 3.79% for MoS_2_, MoSe_2_, MoTe_2_, WS_2_, and WSe_2_ heterostructures, respectively, in Fig. 1. Such small values of lattice mismatch indicates that the total strains will lead to moderately small effects on the electronic structure of the heterostructures.

**Fig. 2:**
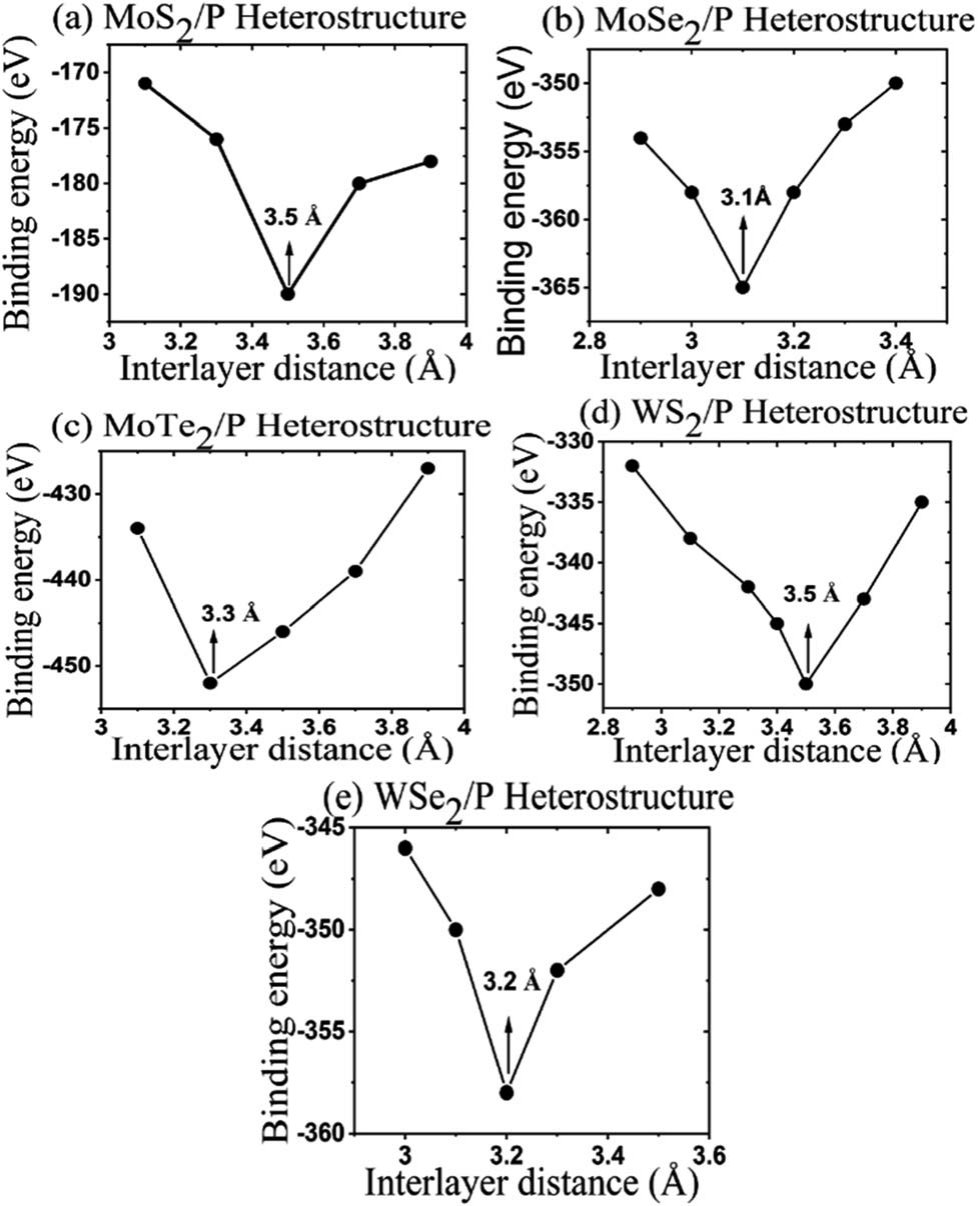
Graph of binding energy *vs.* the distance of interlayer.

The binding energy at the heterostructure interface is calculated for obtaining the most stable configuration of the heterostructure. The binding energy at the interface is defined as *E*_B_ = {*E* − (*E*_1_ + *E*_2_)}/*N*, where *E* is the total energies of MoS_2_/P, MoSe_2_/P, MoTe_2_/P, WS_2_/P_,_ and WSe_2_/P heterostructure, *E*_1_ is the total energy of free-standing MoS_2_, MoSe_2_, MoTe_2_, WS_2_, WSe_2_, and *E*_2_ is the total energy of phosphorene monolayer. N represents the total number of atoms in the heterostructure.^65^ The calculated binding energies of the heterostructures *versus* interlayer distance between the monolayers of MoS_2_, MoSe_2_, MoTe_2_, WS_2_, WSe_2_, and phosphorene are shown in Fig. 2.

For obtaining stable configurations of heterostructure, binding energies of the structures should be negative, which is obtained in Fig. 2 for all the considered heterostructures. The interlayer gap at equilibrium for MoS_2_/P, MoSe_2_/P, MoTe_2_/P, WS_2_/P, and WSe_2_/P are calculated as 3.5 Å, 3.1 Å, 3.3 Å, 3.5 Å, 3.2 Å, respectively with per atom binding energy of about 6820.934 eV, 7096.603 eV, 14 522.7597 eV, 6647.294 eV, and 6925.11452 eV respectively. Such values of binding energies shows that resultant heterostructures have strong van der Waals interaction.

### B. Optical properties

The optical properties and dielectric constant of the TMD heterostructures are evaluated. The performance of the dielectric material in terms of the electric energy storage is expressed by its dielectric constant (*ε*). The behavior of incident light when passing through a material is defined by its dielectric constant.

**Fig. 3:**
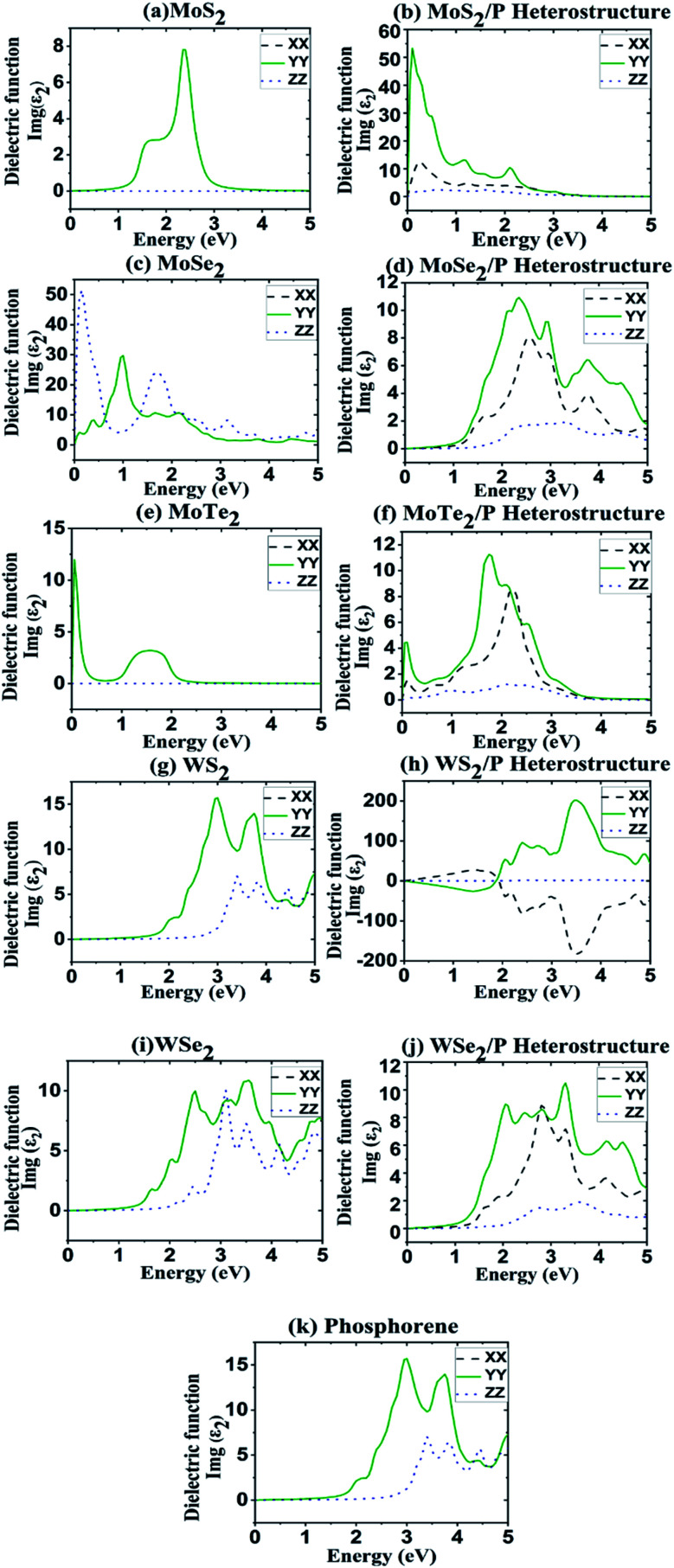
Imaginary parts of dielectric constant along the XX, YY and ZZ tensors as a function of energy for TMDs and their heterostructures with phosphorene. (a) MoS_2_ (c) MoSe_2_, (e) MoTe_2_, (g) WS_2_ and (i) WSe_2_. Heterostructures of (b) MoS_2_/P, (d) MpSe_2_/P, (f) MoTe_2_/P, (h) WS_2_/P and (j) WSe_2_/P. Dielectric function of phosphorene is shown in (k).

The polarization and abnormal dispersion effects are resolved by the real part of dielectric constant, while neutral charge excitation oriented energy absorption in a system is measured by the imaginary part. Fig. 3 shows the imaginary (*ε*_2_) components of the calculated dielectric function as part of photon energy along *XX*, *YY* and *ZZ* directions for the heterostructures.

For finding the optical absorption of 2D materials, the dielectric function's imaginary part (*ε*_2_) is an important parameter, thus we consider only the curves of *ε*_2_ in *y*-direction, whereas the results of remaining two directions can be deduced from the same.^66^ In Fig. 3, for MoS_2_ monolayer, there are two peaks: the strongest at ∼2.5 eV and the sub-strongest at ∼1.8 eV.

**Fig. 4:**
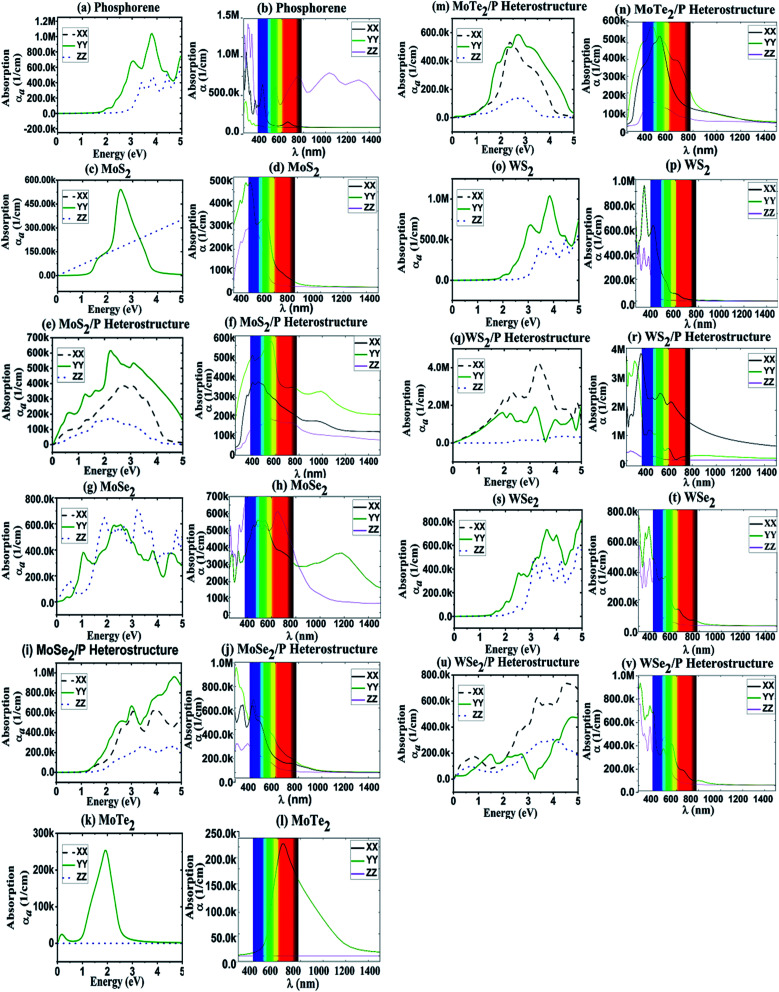
Absorption coefficient as a function of energy for (a) phosphorene, (c) MoS_2_, (g) MoSe_2_, (k) MoTe_2_ (o) WS_2_, (s) WSe_2_, and heterostructures of (e) MoS_2_/P, (i) MoSe_2_/P, (m) MoTe_2_/P, (q) WS_2_/P and (u) WSe_2_/P along the XX, YY and ZZ tensors. Absorption coefficient as a function of wavelength are also shown for (b) phosphorene, (d) MoS_2_, (h) MoSe_2_, (l) MoTe_2_, (p) WS_2_, (t) WSe_2_, and heterostructures of (f) MoS_2_/P, (j) MoSe_2_/P, (n) MoTe_2_/P, (r) WS_2_/P and (v) WSe_2_/P along the XX, YY and ZZ tensors.

Similarly, dielectric peaks for other TMD monolayers can be observed in Fig. 3. For pristine phosphorene, most of the peaks are in the range of 2.5–4.0 eV with the strongest peak appearing at ∼3 eV. The peaks in absorption coefficient in Fig. 4 are in course with peaks in dielectric function. These range of dielectric function values obtained for MoS_2_, MoSe_2_, MoTe_2_, WS_2_, WSe_2_ and phosphorene are matching the values reported in ref. 66 and 67. Whereas, for TMD heterostructures of MoS_2_/P, MoSe_2_/P, MoTe_2_/P, WS_2_/P, and WSe_2_/P, major peaks in the dielectric function are obtained in the region 0–2 eV, 1.5–4.5 eV, 1.5–2.5 eV, 2–4.5 eV, 1.5–4.5 eV, respectively.

From Fig. 3 and 4, it can be observed that the dielectric and absorption edges for all the structures are matching their bandgaps in Fig. 6. In the absorption coefficient plot of Fig. 4, a shift in absorption edges towards lower energy range has been seen for the heterostructures. The existence of the redshift phenomenon can also be observed in all the heterostructures in comparison to absorption in pristine phosphorene. It can be observed from Fig. 4 that phosphorene has very high absorption peaks in the UV region (100–380 nm range), and here TMD based heterostructures have been investigated for tuning/shifting these absorption peaks of phosphorene from UV to visible (from left to right/from high energies to low energies) region or in other words shifting the absorption peaks from UV to red-end of the visible region in the spectrum (red-shift).

This shifting of absorption towards lower-energy (red-shift) is due to the fact that the heterostructures have a lower bandgap because of which the photo-generated electrons can be excited directly from the valence band of TMDs to the conduction band of phosphorene thereby resulting in larger absorption in the visible region. In heterostructures, peaks in refractive index also appear to shift towards lower energies in comparison to the peaks in phosphorene, which also justifies that more photons will be absorbed in the visible region or a resulting red-shift which is confirmed my absorption peaks in this region in Fig. 4. This means that the heterostructures would result in strong and stable absorption of light within the visible range in comparison to individual TMDs or phosphorene. The existence of shift phenomenon is a result of lower bandgap in the heterostructure due to which the conduction band of phosphorene is directly filled by the excited photo-generated electrons of the valence band of MoS_2_, MoSe_2_, MoTe_2_, WS_2_, and WSe_2_. Hence, it can be said that for visual light the heterostructure would display aggravated absorption.

Since dielectric constant is directly proportional to absorption coefficient, peaks in the dielectric function in Fig. 3 convert into the peaks in the absorption coefficient in Fig. 4. In MoS_2_, MoSe_2_, MoTe_2_, WS_2_, and WSe_2_, strongest peaks can be observed at around 2.5 eV, 2.4 eV, 2 eV, 3.8 eV and 5 eV, respectively. Whereas, there are two peaks located at around 3 eV and 3.8 eV for phosphorene. The dielectric constant values are in consistence with values of absorption coefficient. The heterostructure results in stable broader spectrum range of absorption in comparison to its constituent layers, which can be useful in optoelectronic applications.

**Fig. 5:**
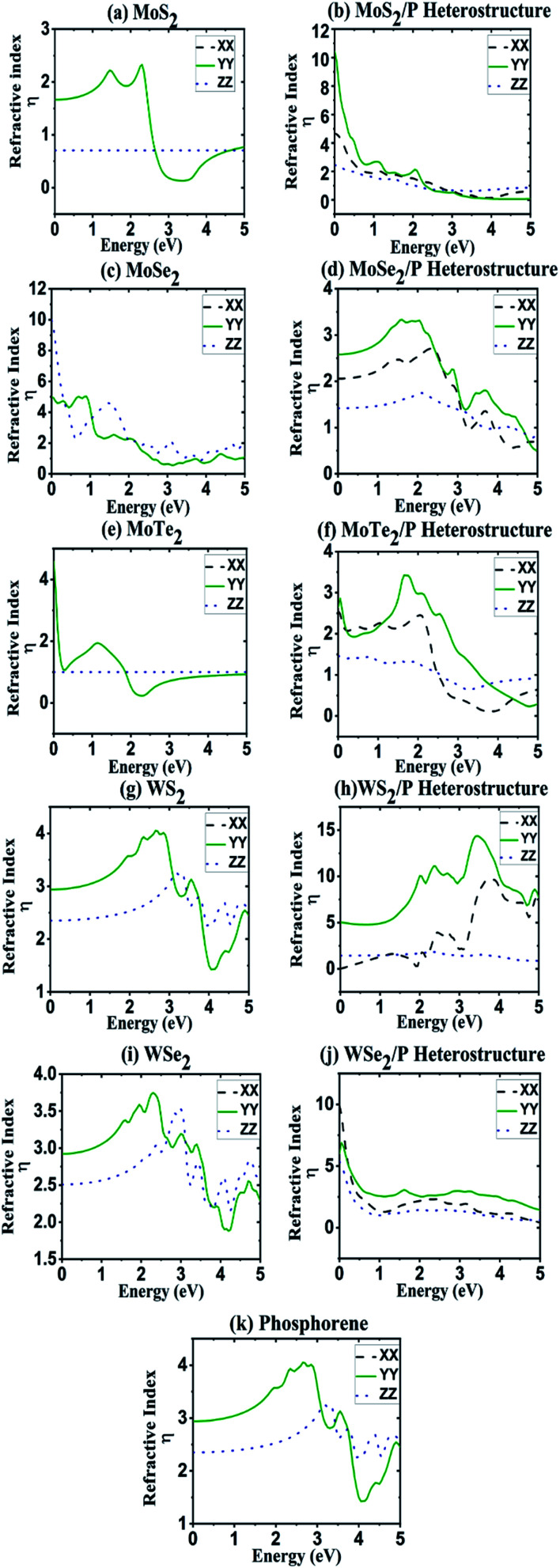
Refractive index as a function of energy for (a) MoS_2_, (c) MoSe_2_, (e) MoTe_2_ (g) WS_2_, (i) WSe_2_ and (k) phosphorene. Refractive index of heterostructures of (b) MoS_2_/P, (d) MoSe_2_/P, (f) MoTe_2_/P, (h) WS_2_/P and (j) WSe_2_/P along the XX, YY and ZZ tensors.

Furthermore, it is noticed that in MoS_2_, MoSe_2_, MoTe_2_, WS_2_, and WSe_2_, YY and XX tensors overlap one another, while the ZZ tensor is free from overlapping. This implies anisotropic or isotropic behavior of monolayer MoS_2_, MoSe_2_, MoTe_2_, WS_2_, and WSe_2_, as in ref. 67. It is observed from Fig. 3–5 that the anisotropy property of phosphorene has been carried over in phosphorene based TMD heterostructures.

Refractive indexes (*η*) are computed from the dielectric functions for all the structures and are shown in Fig. 5. The peaks in refractive index follows the trends in both dielectric constant and absorption coefficient. The heterostructures of MoS_2_/P, MoSe_2_/P, MoTe_2_/P, WS_2_/P, and WSe_2_/P with their major peaks in the refractive index are obtained in the region 0–0.2 eV, 1.2–2.2 eV, 1.5–2.1 eV, 3.0–4.0 eV, and 0–0.2 eV, respectively. The peaks in refractive index in Fig. 5 for all the heterostructures corresponds to the peaks in dielectric constant in Fig. 3. The regions in Fig. 5 where refractive index is large would result in large absorption as it means light would stay for a longer time in these specific regions. Similarly, the portions where refractive index is small would result in lower absorption. The light is expected to stay longer in the systems with larger refractive index, resulting due to larger absorption of the photons in such systems. Lower absorption is observed in some regions, despite having high dielectric constant values in those regions. This reduction in absorption is due to lower values of refractive index in those regions, which is in agreement with the dielectric function (*ε*_2_) for the particular structures.

### C. Electronic structure

**Fig. 6:**
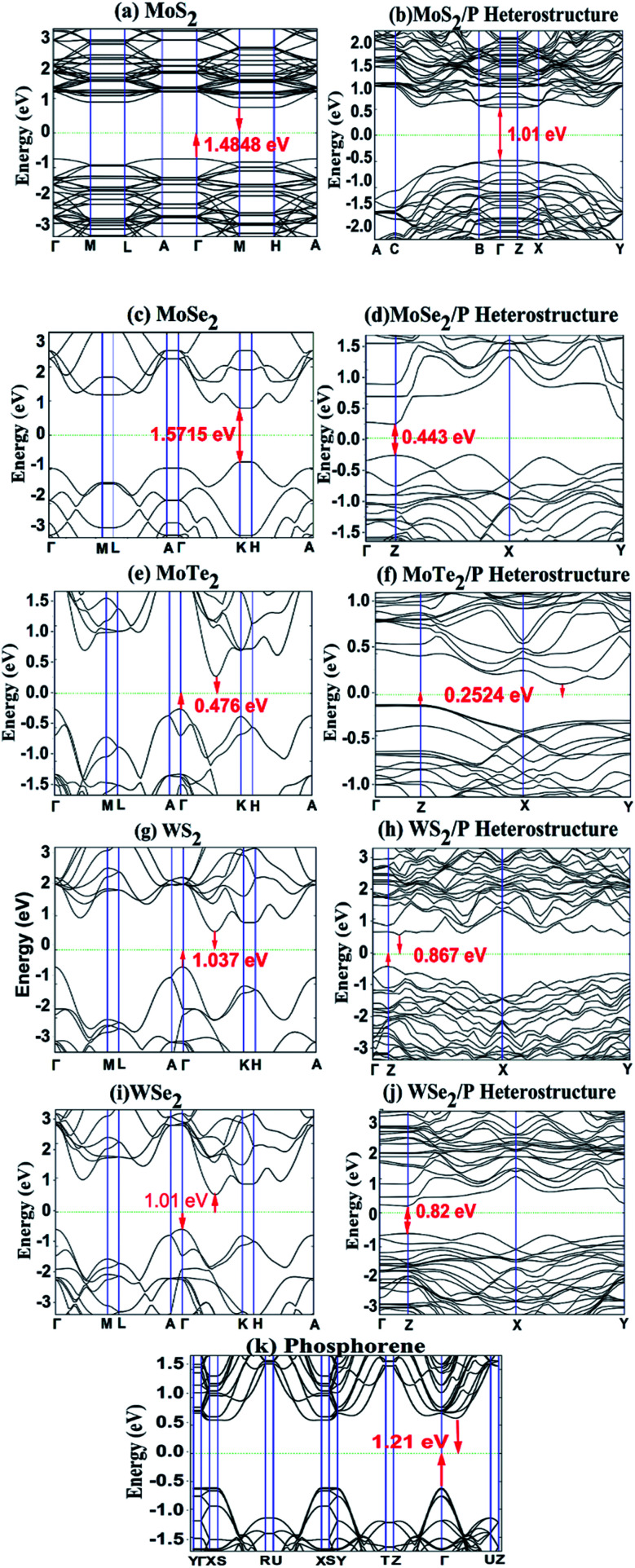
Band structure of (a) MoS_2_, (c) MoSe_2_, (e) MoTe_2_ (g) WS_2_, (i) WSe_2_ and (k) phosphorene, and heterostructures of (b) MoS_2_/P, (d) MoSe_2_/P, (f) MoTe_2_/P, (h) WS_2_/P and (j) WSe_2_/P, with their bandgaps.

For investigating the electronic structure, we calculated band structure of the monolayer MoS_2_, MoSe_2_, MoTe_2_, WS_2_, WSe_2_ and phosphorene, and also the band structures of heterostructures of MoS_2_/P, MoSe_2_/P, MoTe_2_/P, WS_2_/P, and WSe_2_/P for a comparative study. The band structures are shown in Fig. 6. It can be observed from Fig. 6 that the phosphorene monolayer has a direct bandgap of ∼1.21 eV. Whereas, for MoS_2_, MoSe_2_, MoTe_2_, WS_2_, and WSe_2_ monolayer, gap between the conduction band minima (CBM) and valence band maxima (VCM) is observed as bandgap of value 1.48 eV, 1.57 V, 0.47 eV, 1.037 eV and 1.01 eV, respectively. All these values of calculated bandgaps match well with the values reported in previous studies.^17,45,56^ It is interesting to observe from Fig. 6 that the heterostructures of MoS_2_/P, MoSe_2_/P, MoTe_2_/P, WS_2_/P, and WSe_2_/P show indirect bandgap of 1.01 eV, 0.443 eV, 0.2524 eV, 0.867 eV and 0.82 eV, respectively.

### D. Band alignment

**Fig. 7:**
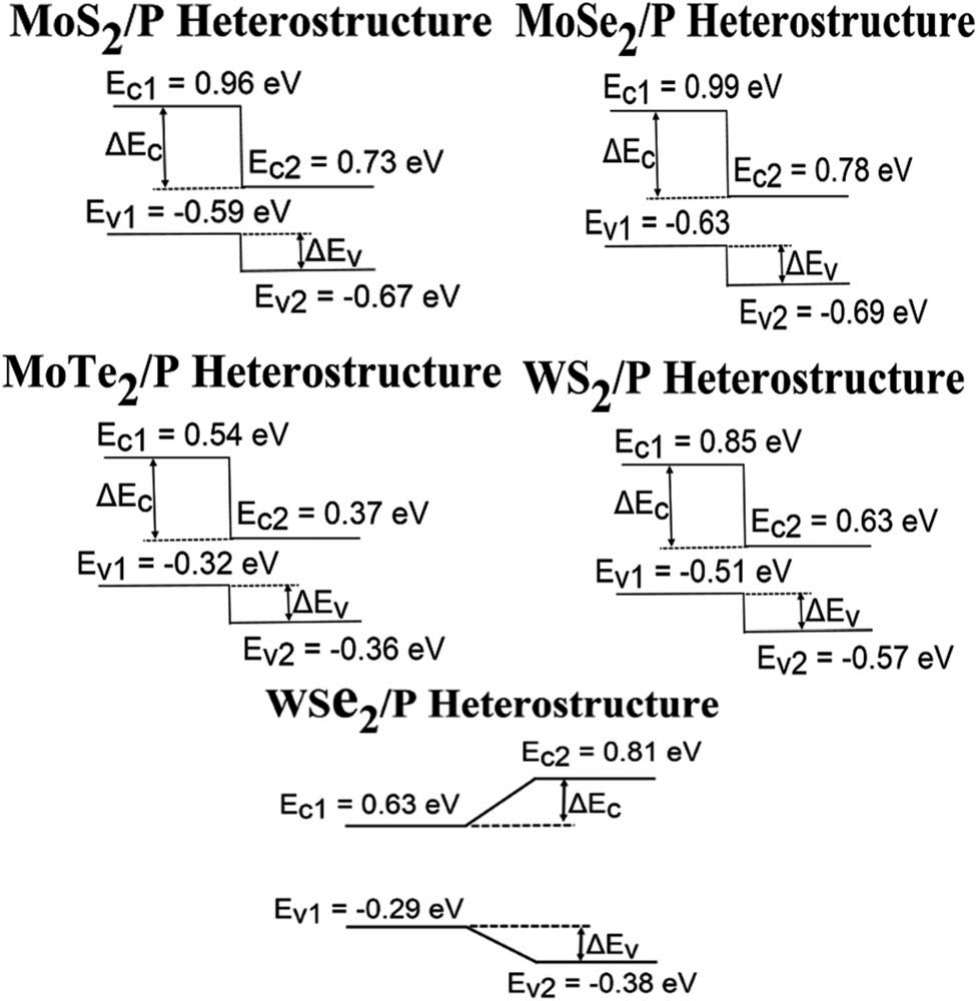
Band alignment of resultant TMD/phosphorene heterostructures.

It is important know the type of band alignment in a material for its use in optical and solar cell applications. Band alignment is computed in Fig. 7 using the valence band and conduction band offsets from their heterostructure interface calculations.^68^

From Fig. 7, it can be observed that all the heterostructures are having type-II band alignments except WSe_2_/P heterostructure. This type of band alignment is important as it helps to separate electron and holes in a natural way, which makes the heterostructures excellent for application in optoelectronics and solar energy conversion.^61^

Further moving away from heterostructure interface to the region where only monolayer of MoS_2_/MoSe_2_/MoTe_2_/WS_2_/WSe_2_ is present, a small band bending is observed in all the structures. The difference between the heterostructure's potential (work function (*W*) and the monolayer's potential (*W*_s_) gives the value of this band bending: Δ*E*_f_ = *W* − *W*_s_. The band bending values for MoS_2_, MoSe_2_, MoTe_2_, WS_2_ and WSe_2_ obtained are 0.16, 0.18, 0.56, 0.13 and 0.12, respectively, and for phosphorene it is −0.998. This suggests that electrons transition will be taken place from MoS_2_/MoSe_2_/MoTe_2_/WS_2_/WSe_2_ to phosphorene. This happens because when two layers with distinct work functions are stacked, then there exists charge transfer from both sides to neutralize the work function differences between the two layers.

## IV. Conclusions

In summary, first principle calculations are performed to study the stacking effect on optoelectronic properties of the heterostructures of MoS_2_/P, MoSe_2_/P, MoTe_2_/P, WS_2_/P, and WSe_2_/P. The results suggest a wide usable spectrum in the visible region with high absorption coefficient values in this region in the heterostructures. Higher visible region absorption is seen in the heterostructures as compared to the monolayers. Also, these results are in confirmation with the trends in dielectric constants and refractive index. The results suggest a large improvement in desired visible region absorption, thereby, increasing the usefulness of TMD based phosphorene heterostructures in solar energy conversion applications and optoelectronic devices.

## Conflicts of interest

There are no conflicts to declare.

## Supplementary Material
